# PARP14 Contributes to the Development of the Tumor-Associated Macrophage Phenotype

**DOI:** 10.3390/ijms25073601

**Published:** 2024-03-22

**Authors:** Isotta Sturniolo, Csongor Váróczy, Zsolt Regdon, Anett Mázló, Szabolcs Muzsai, Attila Bácsi, Giorgia Intili, Csaba Hegedűs, Mark R. Boothby, Jacob Holechek, Dana Ferraris, Herwig Schüler, László Virág

**Affiliations:** 1 Department of Medical Chemistry, Faculty of Medicine, University of Debrecen, 4032 Debrecen, Hungary; isotta.sturniolo@med.unideb.hu (I.S.); csongor.varoczy@gmail.com (C.V.); regdike1988@gmail.com (Z.R.); hcsaba@med.unideb.hu (C.H.); 2 Doctoral School of Molecular Medicine, University of Debrecen, 4032 Debrecen, Hungary; 3 National Academy of Scientist Education, 4032 Debrecen, Hungary; 4 Department of Immunology, Faculty of Medicine, University of Debrecen, 4032 Debrecen, Hungary; anett.mazlo@gmail.com (A.M.); muzsai.szabolcs@med.unideb.hu (S.M.); bacsi.attila@gmail.com (A.B.); 5 Gyula Petrányi Doctoral School of Clinical Immunology and Allergology, University of Debrecen, 4032 Debrecen, Hungary; 6 HUN-REN-DE Allergology Research Group, 4032 Debrecen, Hungary; 7 Department of Biomedicine, Neuroscience and Advanced Diagnostics (BIND), University of Palermo, 90133 Palermo, Italy; giorgia.intili@unipa.it; 8 Department of Pathology, Microbiology and Immunology, Vanderbilt University, Nashville, TN 37235, USA; mark.boothby@vumc.org; 9 Agilent Technologies, Wilmington, DE 19808, USA; jake.holechek@agilent.com; 10 Department of Chemistry, McDaniel College, Westminster, MD 21157, USA; dferraris@mcdaniel.edu; 11 Center for Molecular Protein Science, Department of Chemistry, Lund University, 22100 Lund, Sweden; herwig.schuler@biochemistry.lu.se; 12 HUN-REN-DE Cell Biology and Signaling Research Group, 4032 Debrecen, Hungary

**Keywords:** macrophage, ADP-ribosylation, breast cancer, PARP14

## Abstract

Cancers reprogram macrophages (MΦs) to a tumor-growth-promoting TAM (tumor-associated MΦ) phenotype that is similar to the anti-inflammatory M2 phenotype. Poly(ADP-ribose) polymerase (PARP) enzymes regulate various aspects of MΦ biology, but their role in the development of TAM phenotype has not yet been investigated. Here, we show that the multispectral PARP inhibitor (PARPi) PJ34 and the PARP14 specific inhibitor MCD113 suppress the expression of M2 marker genes in IL-4-polarized primary murine MΦs, in THP-1 monocytic human MΦs, and in primary human monocyte-derived MΦs. MΦs isolated from PARP14 knockout mice showed a limited ability to differentiate to M2 cells. In a murine model of TAM polarization (4T1 breast carcinoma cell supernatant transfer to primary MΦs) and in a human TAM model (spheroids formed from JIMT-1 breast carcinoma cells and THP-1-MΦs), both PARPis and the PARP14 KO phenotype caused weaker TAM polarization. Increased JIMT-1 cell apoptosis in co-culture spheroids treated with PARPis suggested reduced functional TAM reprogramming. Protein profiling arrays identified lipocalin-2, macrophage migration inhibitory factor, and plasminogen activator inhibitor-1 as potential (ADP-ribosyl)ation-dependent mediators of TAM differentiation. Our data suggest that PARP14 inhibition might be a viable anticancer strategy with a potential to boost anticancer immune responses by reprogramming TAMs.

## 1. Introduction

Breast cancer is the most common malignant tumor in women, while 1–2% of cases occur in men. The tumor develops in the milk ducts of the breast or in the milk-producing gland tissue. According to the WHO, more than 2 million new cases are reported each year and close to 700,000 women die from the disease every year [1]. Traditional treatment options include surgical removal of the tumor, radiotherapy, and cytotoxic chemotherapy. In addition, based on the cancer marker profile of the tumor with special regard to hormone (estrogen and progesterone) receptor expression and overexpression of the *HER-2* oncogene, further treatment modalities such as hormone therapy and monoclonal antibody therapy may further improve the outcome of the disease [2].

It has been increasingly recognized that cells of the tumor stroma strongly influence tumor behavior, tumor cell proliferation, and resistance to therapies [3]. Various immune cells of the innate and adaptive immune system are an integral part of the tumor stroma and can have both positive and negative effects on the disease outcome [4]. The first line of defense of the immune system is the innate immune response (macrophages, dendritic cells, granulocytes, mast cells, NK and NKT cells, etc.). As for the adaptive immune system, CD8^+^ T lymphocytes and B-lymphocytes, as well as CD4^+^ T cells regulating both lines of adaptive immunity, are central parts of the tumor-associated immune network [5]. The immune landscape of breast cancer is also rich in immune cells [6]. However, a strong presence of immune cells in the tumor does not necessarily mean a robust anti-tumor immune response. In fact, most tumors send suppressive signals to immune cells and can reprogram them to a tolerant or even a tumor-growth-promoting phenotype [7]. Novel immunotherapies of cancer (e.g., immune checkpoint inhibitors) aim at disrupting tumor-induced immunosuppressive signaling or equip effector immune cells (e.g., cytotoxic T cells) with tumor antigen recognition tools (e.g., chimeric antigen receptors, CARs) to enhance their tumor-cell-killing activities [8,9]. The remarkable clinical efficiency of immune checkpoint inhibitors and CAR-T cells proved that, contrary to previously held beliefs, antitumor immune responses can be successfully unleashed in breast cancer [10].

Macrophages (MΦs) are present in most tumors, and they often represent the most dominant immune cells in tumors. However, the presence of MΦs in the tumor stroma is mostly viewed as a sign of bad prognosis [11,12]. MΦs are highly plastic innate immune cells. In response to stimuli from the environment, they can adopt a wide range of phenotypes, ranging from the so-called inflammatory (M1) phenotype to the anti-inflammatory (M2) phenotype that mediates the resolution of inflammation [13]. Tumor-associated macrophages (TAMs) display a characteristic phenotype resembling M2 cells [14]. It has been proposed that reprogramming TAMs from M2-like behavior to the M1-like phenotype may provide a therapeutic benefit in cancer treatment [13].

ADP-ribosylation is a posttranslational protein modification that regulates various cellular functions in many cell types, including MΦs [15]. ADP-ribosyl transferase (ADPRT) also known as poly(ADP-ribose) polymerase (PARP) enzymes belong to a 17 member ADPRT/PARP enzyme family. All of these enzyme can cleave NAD+ into nicotinamide and ADP-ribose and can covalently attach the latter to various target proteins, a process called mono(ADP-ribosyl)ation (MARylation) [16,17]. Some members of the enzyme family (e.g., PARP1-3) can add further ADP-ribose units to the first one and synthesize a poly(ADP-ribose) (PAR) polymer onto target proteins (PARylation) [15]. ADP-ribosylation has been shown to regulate a wide variety of cellular functions ranging from DNA repair, genomic stability, telomere maintenance, and mitosis to gene transcription, RNA degradation, translation, and cell death [18]. Of note, PARP1 has been shown to contribute to inflammatory gene expression mainly via acting as a co-activator of *NFκB* [19]. Other PARPs, such as PARP9 and PARP14, have been reported to cross-regulate M1 and M2 polarization of MΦs in the context of coronary atherosclerosis [20]. Although PARP14 has received less attention compared to the other enzymes of the PARP family, it has currently gained recognition as a promising target for treating chronic inflammation. Recent publications have also demonstrated the tumor-promoting activities of PARP14 [21,22,23,24]. However, despite being recognized as an oncoprotein, there is limited information available regarding the specific roles of PARP14 in the context of tumor immune evasion, and little is known about the role of ADP-ribosylation in the polarization of TAMs. Here, we set out to investigate how the inhibition of ADP-ribosylation affects the TAM phenotype and show that PARP14 plays a key role in breast cancer-induced MΦ polarization.

## 2. Results

### 2.1. PARP14 Mediated M2 Polarization in Murine BMDMs

**Figure 1:**
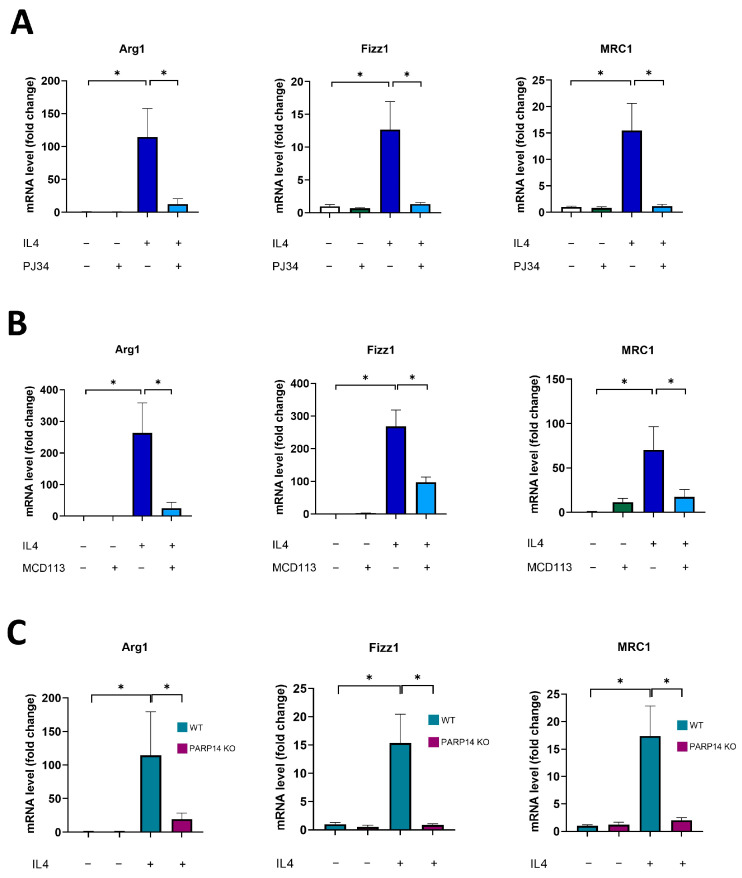
PARP14 mediated M2 polarization in BMDMs. Bone-marrow-derived macrophages (BMDMs) from C57/BL6 mice were differentiated with 25 ng/mL M-CSF for 6 days. BMDMs were pre-treated with either 20 μM PJ34 (**A**) or 50 μM MCD113 (**B**) for 1 h and then polarized into M2 macrophages with 5 ng/mL IL-4 for 24 h. On day 7, mRNA levels of the M2 markers *Arg1*, *Fizz1*, and *MRC1* were determined by RT-qPCR. BMDMs were also differentiated from PARP14 KO mice and their wild type counterparts with 25 ng/mL M-CSF for 6 days and then polarized into M2 with 5 ng/mL IL-4 for 24 h. RNA extraction was performed on day 7 followed by RT-qPCR (**C**). Light blue and purple colors in (**C**) represent WT and PARP14 KO groups, respectively. The graphs show the mean of at least 3 independent experiments (±SEM). Statistical evaluation was performed with the Kruskal–Wallis test followed by Dunn’s post-hoc test (**A**) (*Arg1* in (**B**)) or with one-way ANOVA and Tukey’s post-hoc test (**C**) (*Fizz1* and *MRC1* in (**B**)) (* *p* < 0.05).

First, we established an in vitro murine 2D cell culture model to investigate M2 polarization. Bone-marrow-derived macrophages from C57/BL6 mice were differentiated with M-CSF for 6 days and were polarized to the M2 phenotype with IL-4 [25]. To characterize the functional state of macrophages, a panel of M2 markers was used [26], and their mRNA levels were quantified by RT-qPCR. As expected, IL-4 treatments triggered the increase in all M2 marker genes, namely, Arginase1 (*Arg1*), *Fizz1*, and Mannose Receptor 1 (*MRC1*) (Figure 1).

A key question we aimed to address was the role of PARylation and certain individual PARP enzyme family members in MΦ polarization. In line with a recent study [27], our preliminary experiments with the clinically used PARP1/2-specific, narrow-spectrum PARP inhibitor (PARPi) Olaparib indicated that PARP1 is not likely to be involved in M2 polarization. However, a relatively high concentration of another wide-spectrum PARPi, PJ34, significantly inhibited all the M2 markers studied (Figure 1A). By comparing the chemical space of these inhibitors, it was reported that PJ34 but not Olaparib inhibits PARP14 [28], an enzyme that has been implicated in IL-4 response [20,29]. Thus, our hypothesis was that PARP14 mediates M2 polarization of MΦs. Therefore, we tested the PARP14 inhibitor MCD113 (a kind gift from Dana Ferraris, McDaniel College) [30] in our model and found that it also suppressed M2 marker gene expression (Figure 1B). To further strengthen the role of PARP14 in MΦ polarization, we repeated the M2 polarization experiments with BMDM-derived MΦs isolated from PARP14KO mice [31]. Our RT-qPCR data showed that IL-4-stimulated PARP14^−/−^ MΦs did not polarize toward the M2 phenotype and did not express the M2 markers (Figure 1C). Taken together, these data indicate that PARP14 plays an essential role in M2 polarization.

### 2.2. Soluble Factors Produced by 4T1 Murine Breast Cancer Cells Reprogrammed Murine BMDMs toward an M2-like TAM Phenotype in a PARP14-Dependent Manner

**Figure 2:**
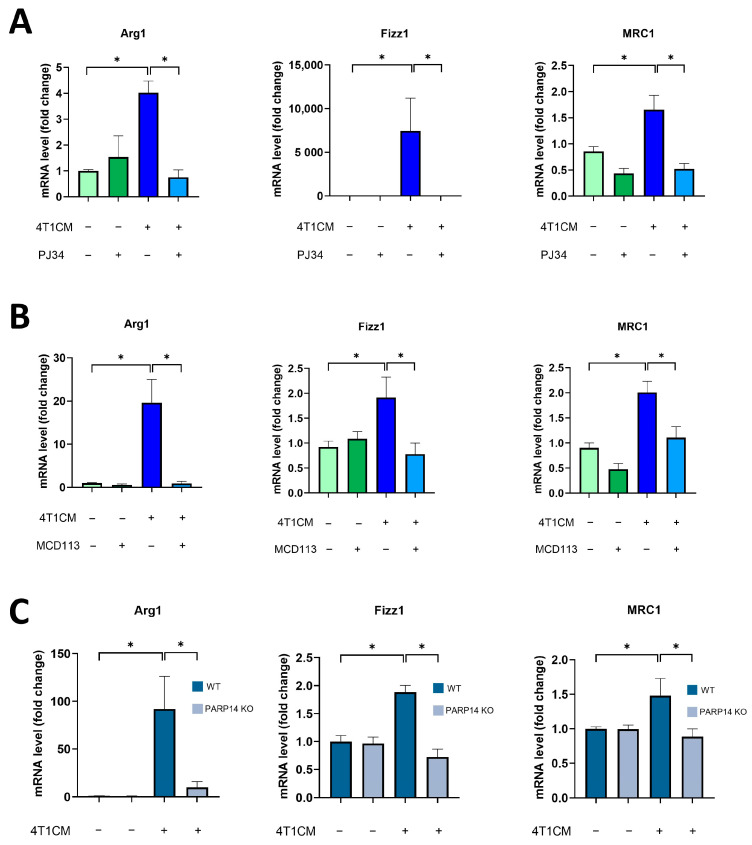
Soluble factors produced by 4T1 murine breast cancer cells reprogrammed murine BMDMs toward an M2-like TAM phenotype in a PARP14-dependent manner. Bone-marrow-derived macrophages (BMDMs) were differentiated from C57/BL6 mice with 25 ng/mL M-CSF for 6 days. Cells were pre-treated with either 20 μM PJ34 (**A**) or 50 μM MCD113 (**B**) for 1 h and then treated with 4T1 conditioned media (4T1CM; diluted at a 1:1 ratio with full DMEM) for 24 h to polarize them into TAMs. On day 7, mRNA levels of M2 markers were quantified by RT-qPCR. BMDMs were also differentiated from PARP14 KO mice and their wild-type counterparts with 25 ng/mL M-CSF for 6 days and then treated with 4T1CM for 24 h. RNA extraction was performed on day 7 followed by RT-qPCR (**C**). Dark blue and grey colors in (**C**) represent WT and PARP14 KO groups, respectively. The graphs show the mean of at least 3 independent experiments (±SEM). Data were analyzed using the Kruskal–Wallis test and Dunn’s post-hoc test (**A**,**B**) and with one-way ANOVA followed by Tukey’s post-hoc test (**C**) (* *p* < 0.05).

To investigate the role of PARP14 in TAM polarization, we set up an in vitro model in which the supernatant (or conditioned media, CM) was transferred from cancer cell lines to MΦs. BMDMs were differentiated from C57/BL6 mice with M-CSF for 6 days, as described in Section 4, and then cultured with conditioned media collected from the 4T1 mouse mammary carcinoma cells and diluted at a 1:1 ratio with complete DMEM medium [32,33] for 24 h. We observed that the conditioned media (CM) of 4T1 cells (4T1CM) induced M2-like polarization in BMDMs as indicated by M2 marker (*Arg1*, *Fizz1*, and *MRC1*) expression (Figure 2). To assess the role of PARylation in the polarization of TAMs, we used the PARPi PJ34 and the PARP14i MCD113 in this model. Interestingly, we found that both inhibitors significantly reduced the expression of the M2 markers, suggesting that ADP-ribosylation via PARP14 contributes to the induction of the TAM phenotype (Figure 2A,B). The absence of PARP14 in BMDMs isolated from PARP14^−/−^ mice also resulted in suppression of the 4T1CM-induced TAM phenotype (Figure 2C). Taken together, these data demonstrate that 4T1CM triggers an M2-like polarization of murine BMDMs and suggest that PARP14 is a key mediator of TAM polarization.

### 2.3. PARP14 Mediated M2 Polarization in Human PBMC-MΦs

To find out if PARP14 also plays a key role in the M2 polarization of human MΦs, we used human peripheral blood monocyte (PBMC)-derived macrophages (PBMC-MΦs) differentiated in the presence of M-CSF. PBMC-MΦs were pre-treated with the PARPi PJ34 or the PARP14i MCD113 for 1 h and then stimulated with IL-4 for 24 h to polarize them into the M2 phenotype. On day 6, we confirmed that neither treatment affected the viability of PBMC-MΦs, but their microscopic phenotype changed, as indicated by the formation of cell clusters (Figure S1). Subsequently, cells were collected for RNA extraction, and a detailed expression profile of human M2 markers including *Arg1*, *Fizz1*, *MRC1*, *Fibronectin*, *CCL17*, and *CCL18* was determined by RT-qPCR.

**Figure 3:**
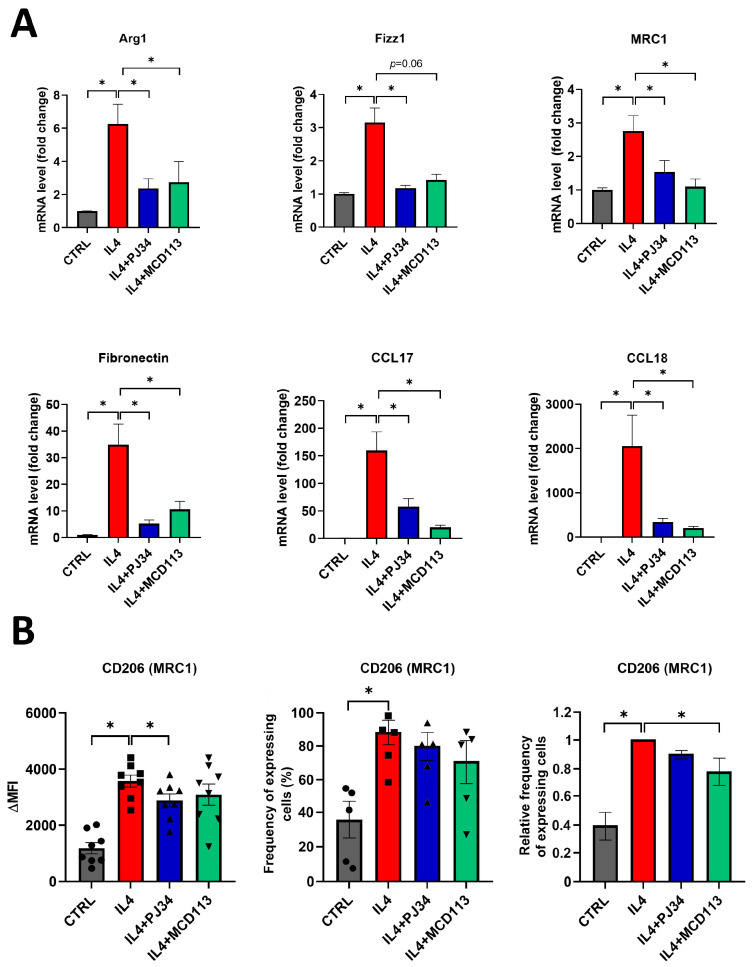
The PARP inhibitor PJ34 and the PARP14 inhibitor MCD113 reduced the expression of M2 markers in human macrophages. Macrophages were differentiated from human monocytes with 50 ng/mL M-CSF for 5 days. On day 5, they were pre-treated with either 20 μM PJ34 or 50 μM MCD113 for 1 h and then stimulated with 100 ng/mL IL-4 for 24 h to polarize them into M2 macrophages. On day 6, cells were collected for RNA extraction, and M2 markers were measured with RT-qPCR. Histograms show the mean of at least 3 independent experiments (±SEM). Statistical evaluation was performed with the Kruskal–Wallis test and Dunn’s post-hoc test (* *p* < 0.05) (**A**). On day 6, cells were also collected and labelled with APC-conjugated anti-human mouse CD206 monoclonal antibody. Fluorescence intensities and the ratio of CD206-expressing cells were measured by flow cytometry. The mean value of MFIs (median fluorescence intensities) of the cell surface molecule CD206 was calculated from 8 independent experiments (±SEM). The mean value of frequency of CD206+ cells was calculated from 5 independent experiments (±SEM). The relative frequency of CD206-expressing cells was determined as follows. For each donor, the frequency of CD206+ cells differentiated in the presence of IL-4 was considered to be 1, and the frequency of untreated cells and cells treated with IL-4 + PARP inhibitors was compared to this. Data were analyzed by one-way ANOVA followed by Bonferroni’s post-hoc test (left panel) or by the Kruskal–Wallis test and Dunn’s post-hoc test (middle and right panels) (* *p* < 0.05) (**B**).

Analyses revealed that all the measured M2 markers were significantly higher in IL-4-stimulated PBMC-MΦs compared to the control, non-polarized cells (Figure 3A).

Furthermore, *Arg1*, *MRC1*, *Fibronectin*, *CCL17*, and *CCL18* mRNA levels were found to be decreased after treatments with either PJ34 or MCD113. Both inhibitors decreased *Fizz1* expression; however, the change was found to be significant only after pre-treatments with PJ34, while it did not reach the level of statistical significance (*p* = 0.06) in the case of MCD113 (Figure 3A).

As the MRC1 (CD206) expression was significantly altered by both PARPis, we examined the appearance of this M2 marker on the surface of PBMC-MΦs and measured the frequency of CD206+ cells. A 1 h treatment of MΦ cells with MCD113 slightly changed the size and granulation of the cells (Figure S1B), but the cells appeared as a well-defined population in flow cytometry. As expected, PBMC-MΦs showed increased CD206 expression upon their polarization with IL-4. In line with the RT-qPCR results, the cell surface expression of CD206 was significantly downregulated by PJ34 pre-treatment and slightly but not significantly reduced upon MCD113 treatment (Figure 3B). Moreover, the relative frequency of CD206-expressing cells was significantly decreased in response to MCD113 treatments (Figure 3B).

These data suggest that the role of PARP14 is similar both in the murine and human models of M2 differentiation.

### 2.4. THP-1-Derived MΦs Incorporated into JIMT-1 Breast Cancer Cell Spheroids Underwent M2-like Polarization

**Figure 4:**
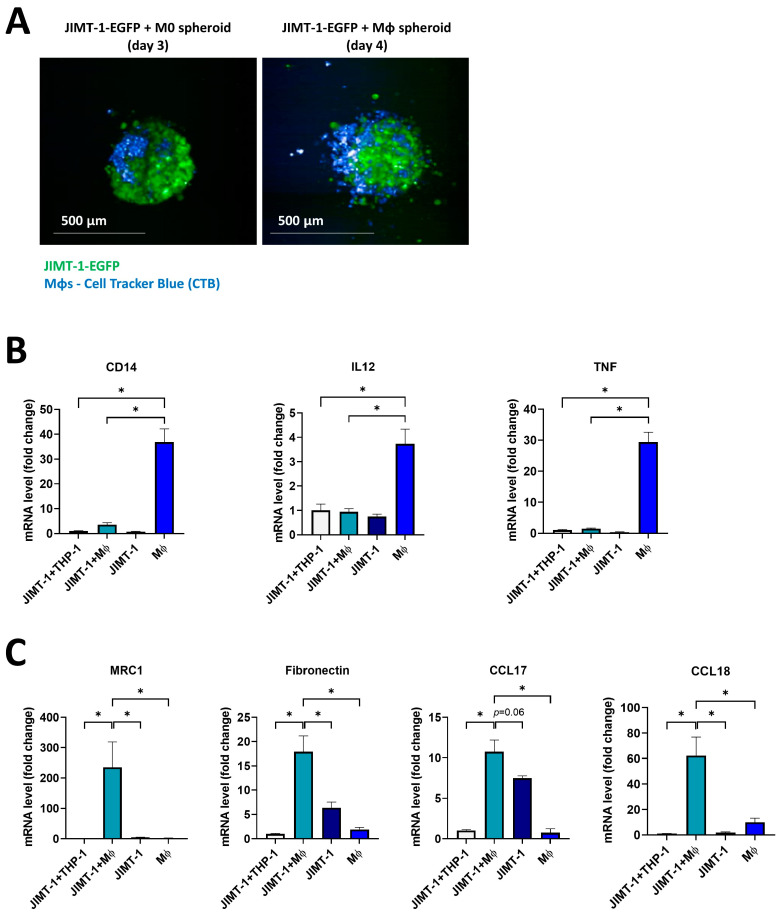
THP-1-derived macrophages incorporated into JIMT-1 breast cancer cell spheroids underwent M2-like polarization. Three-dimensional co-cultures (spheroids) were generated with JIMT-1-EGFP and THP-1-differentiated macrophages (MΦs, differentiated from THP-1 cells with 100 ng/mL PMA for 48 h). JIMT-1-EGFP cells (green) and MΦs (stained with 10 µM Cell Tracker Blue (CTB)) were mixed at a 1:1 ratio. Images were taken on day 3 after the induction of the formation of spheroids (JIMT-1+M0, left) and on day 4 after 20 ng/mL IFN-γ treatments (JIMT-1+MΦ, right) (**A**). mRNA levels of M1 (**B**) and M2 markers (**C**) in spheroids were determined by RT-qPCR. Spheroid co-cultures were kept in cultures for a total time of 4 days (1 day in the presence of IFN-γ). Columns show the relative expression of mRNA levels of the polarization markers in spheroid co-cultures JIMT-1+THP-1, JIMT-1+MΦ, and in JIMT-1 and MΦ spheroids, individually. The graphs show the mean of at least 3 independent experiments (±SEM). Statistical evaluation was performed with the Kruskal–Wallis test followed by Dunn’s post-hoc test, or with one-way ANOVA and Tukey’s post-hoc test (*CD14* in (**B**) and *Fibronectin* in (**C**)) (* *p* < 0.05).

Our next goal was to establish cellular models that more closely resemble in vivo conditions or clinical settings. Hence, we set up an in vitro 3D tumor spheroid model in which cancer cell–MΦ cell interactions could be studied in their complexities to identify the effects of tumor-derived signals and the role of ADP-ribosylation in the differentiation of TAMs. We therefore developed a co-culture spheroid model using human THP-1-derived MΦs [34,35] and the JIMT-1 human breast cancer cell line expressing EGFP. Co-culture spheroids were established as previously described [36]. THP-1-derived MΦs were fluorescently labelled with Cell Tracker Blue (CTB). Microscopic images of spheroids showed that, once differentiated with IFN-γ, MΦs started to infiltrate the spheroids on day 4, although the spheroids did not display any visible change in size after MΦ polarization (Figure 4A).

RT-qPCR assays were run to determine the mRNA levels of a wide panel of M1 (*CD14*, *IL12*, and *TNF*) and M2 (*MRC1*, *Fibronectin*, *CCL17*, and *CCL18*) markers in the co-culture spheroids. Special attention was given to the fact that murine and human MΦ polarization markers differ considerably [13]. First, M1 polarization was induced in the spheroids with IFN-γ. As we could not exclude the possibility that cancer cells also produce some MΦ markers, we compared the gene expression profiles of JIMT-1 cancer cell spheroids with JIMT-1-THP-1 co-culture spheroids. Intriguingly, the gene expression profile analysis demonstrated that MΦ-cancer cell co-cultures displayed significantly decreased levels of M1 markers compared to MΦ spheroids not kept in co-culture (Figure 4B). In addition, the expression levels of M1 markers were always low in the case of JIMT-1 spheroids, confirming that MΦ marker production was generated by MΦs and not the cancer cells. Since TAMs in the tumor microenvironment (TME) display an M2-like phenotype [37,38], we determined M2 marker gene expressions in co-culture spheroids and found that the co-culture with JIMT-1 cells induced the expression of M2 markers in THP-1 cells as compared to “pure” MΦ spheroids (Figure 4C). The findings support our hypothesis that TME signals trigger a polarization of MΦs into an M2-like phenotype (Figure 4C) and are in line with our data obtained with 4T1CM transfer onto mouse MΦs (see above).

Overall, mRNA expression of M1 and M2 markers confirmed the polarization of THP-1-derived MΦs in our 3D system.

### 2.5. The PARPi PJ34 and the PARP14i MCD113 Decreased M2 Marker Gene Expression in JIMT-1-THP-1-Derived MΦ Co-Culture Spheroids

**Figure 5:**
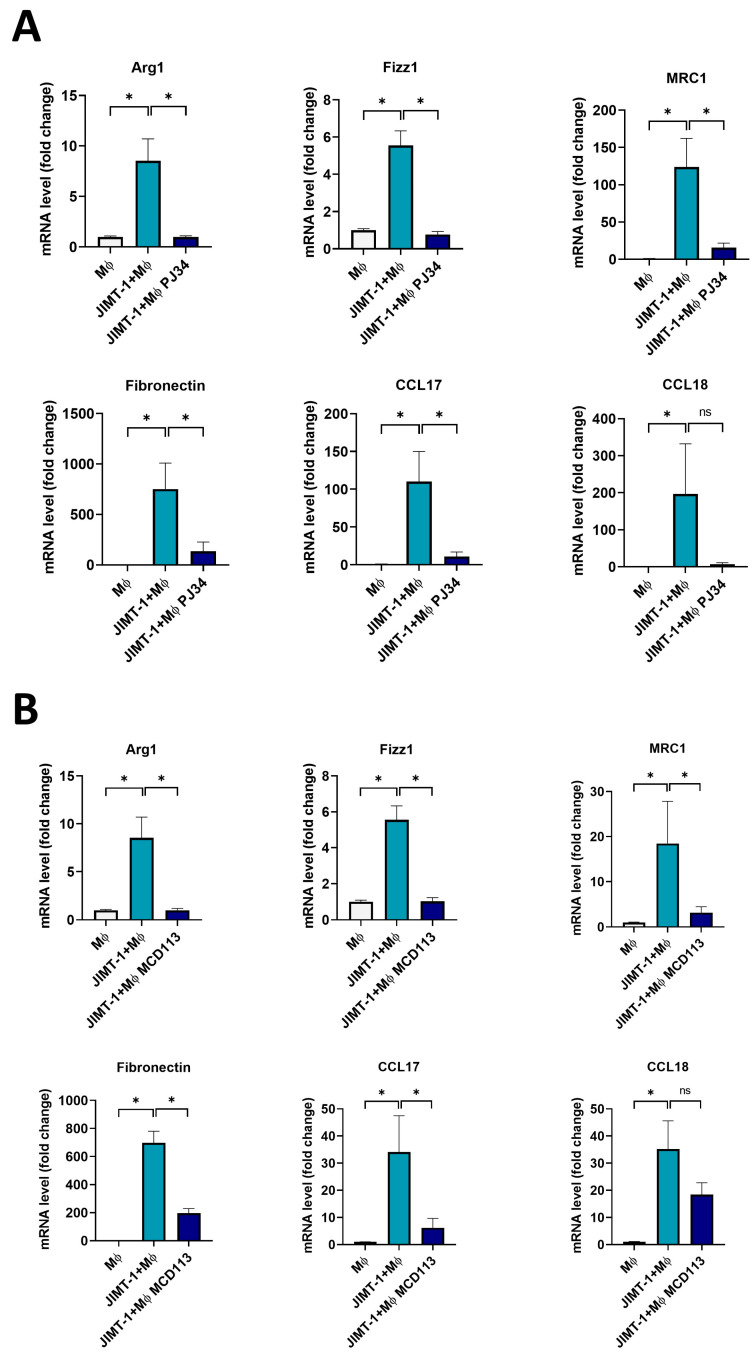
The PARPi PJ34 and the PARP14i MCD113 decreased M2 marker gene expression in JIMT-1-THP-1-derived MΦ co-culture spheroids. Spheroids were grown from co-cultures of JIMT-1 cells and THP-1-derived MΦs or from THP-1-derived MΦs only, as described in Section 4. Spheroids were kept in cultures for 4 days after formation (1 day in the presence of 20 ng/mL IFN-γ), followed by treatments with either 25 μM PJ34 (**A**) or 55 μM MCD113 (**B**) for 24 h. Columns show the relative expression of mRNA levels of M2 markers in spheroids and spheroid co-cultures quantified by RT-qPCR. The histograms represent the mean of at least 3 independent experiments (±SEM). Statistical evaluation was performed with one-way ANOVA and Tukey’s post-hoc test (*MRC1* and *CCL17* in (**A**), *Fibronectin* in (**A**,**B**)), or with the Kruskal–Wallis test and Dunn’s post-hoc test (* *p* < 0.05, ns: not significant).

To investigate the role of ADP-ribosylation in MΦ polarization in 3D co-cultures, spheroids formed in co-cultures of JIMT-1 cells and THP-1-derived MΦs or from pure THP-1-derived MΦs were incubated with PJ34 and MCD113 for 24 h, and M2 marker gene expression was assessed with RT-qPCR. Concentrations of PARPi compounds were slightly increased as cell-to-cell contacts in spheroids are known to lead to cell resistance to toxic stimuli. A wide spectrum of M2 markers was used for the detailed characterization of the functional state of MΦs [26]. Besides *MRC1*, *Fibronectin*, *CCL17*, *CCL18*, *Arg1*, and *Fizz1* were also included in this panel. In line with data presented in Figure 4C, JIMT-1-THP-1 co-cultures stimulated with IFN-γ exhibited significantly higher expression of all M2 markers compared to MΦ-only cultures (Figure 5).

Moreover, co-culture spheroids treated with both the PARPi PJ34 (Figure 5A) and the PARP14i MCD113 showed a significant decrease in the mRNA levels of all M2 markers (Figure 5B). The only exception was the case of *CCL18*, in which there was a visible, but not significant, reduction after incubation with the ADP-ribosylation inhibitor compounds (Figure 5A,B).

### 2.6. Effects of the PARP Inhibitors PJ34 and MCD113 on Morphological Changes and Cell Death of the Spheroid Co-Culture System

**Figure 6:**
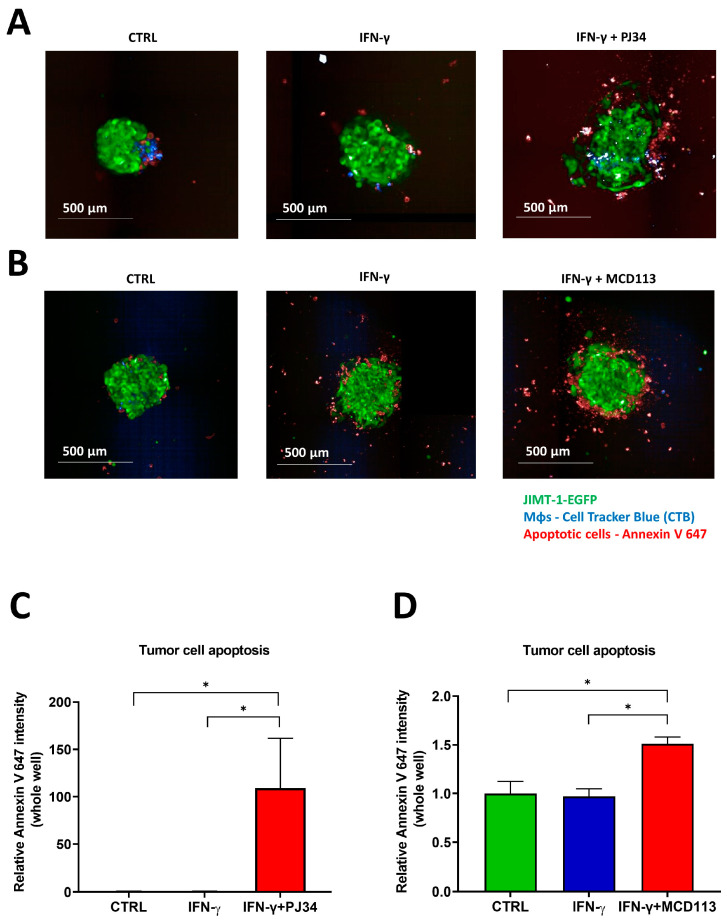
The PARP inhibitors PJ34 and MCD113 induced apoptotic tumor cell death in spheroid co-cultures. Co-culture spheroids were generated with JIMT-1-EGFP (green) and Cell Tracker Blue-stained derived MΦs at a 1:1 ratio. Spheroids were kept in cultures for a duration of 4 days (1 day in the presence of 20 ng/mL IFN-γ) and were then treated with 25 μM PJ34 or 55 μM MCD113 for 24 h. Cells were stained with Annexin V 647 to detect apoptosis. Images were taken after spheroid formation on day 5 following PJ34 and MCD113 treatments ((**A**,**B**) respectively). Images of 3 spheroids/treatment were taken and analyzed for the fluorescence intensity of Annexin V-Alexa 647 in tumor cells/each well (**C**,**D**). Means ± SEM of 3 independent experiments are shown. The statistics were calculated with one-way ANOVA followed by Dunnett’s post-hoc test (* *p* < 0.05).

Afterwards, we investigated the effect of the PARPis PJ34 and MCD113 on (a) the morphology and phenotypic alterations and (b) the tumor cell death in the 3D spheroid co-cultures. Expressing green fluorescent protein (EGFP) JIMT-1 cells co-cultured with THP-1-derived MΦs (stained with CTB) were used to form spheroids. On day 3, they were treated with IFN-γ for 24 h to induce MΦ polarization. On day 4, spheroids were incubated with PJ34 or MCD113 for an additional 24 h. Thereafter, they were stained with Annexin V-Alexa Fluor 647 prior to imaging in order to detect apoptotic cell death. Confocal high-content analysis (HCA) of 3D cultures revealed that, while there was no visible change in size and morphology of spheroids treated with IFN-γ (Figure 6A,B), spheroids co-treated with PJ34 displayed a reduced compactness, and signs of disintegration started to appear in the peripheral region (Figure 6A).

A significant increase in spheroid size was detected after treatments with both PARPi compounds compared to the untreated controls (Figure S2). Moreover, PJ34 and MCD113 treatments caused JIMT-1 cell death in the spheroids, as indicated by the marked elevation of Annexin V positivity (red staining in green cells). Apoptotic (Annexin V-positive) cells indeed emerged in the outer region of the treated spheroids (Figure 6).

These results suggest that ADP-ribosylation-mediated TAM reprogramming may have functional consequences, e.g., the prevention of MΦ-mediated cancer cell killing.

### 2.7. Proteome Profiling Revealed the Possible Roles of LCN2, MIF, and PAI-1 Cytokines in TAM Polarization

**Figure 7:**
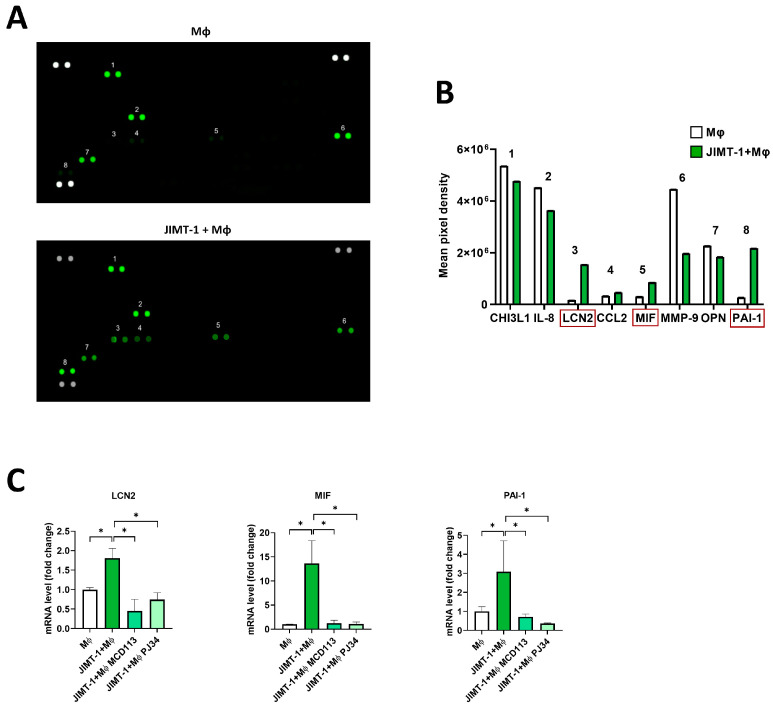
Proteome profiling suggesting the involvement of LCN2, MIF, and PAI-1 in TAM polarization. Spheroids were generated either from THP-1-derived MΦs or JIMT-1-THP-1-MΦ co-cultures. Cells were kept in cultures for 4 days (1 day in the presence of 20 ng/mL IFN-γ), and supernatant was collected. Cell supernatants (500 μL) were added to each membrane array (**A**), and analytes were detected by proteome profiling as described in Section 4 (**B**). Using the same conditions, RNA was also extracted from spheroids, and mRNA levels of the same set of cytokines were quantified by RT-qPCR. Treatments with the PARPi PJ34 (25 μM) and PARP14i MCD113 (55 μM) resulted in a significant decrease in the expression of these cytokines (**C**). The numbers in (**A**,**B**) indicate the different cytokines detected. Red rectangles in (**B**) depict the three cytokines selected for quantitation by RT-qPCR, as shown in (**C**). Graphs show mean of at least 3 independent experiments (±SEM). Statistical evaluation was performed with one-way ANOVA and Tukey’s post-hoc test (* *p* < 0.05).

To further study the mechanisms underlying JIMT-1 cells in promoting the differentiation of TAMs, we performed a human cytokine array to determine the cytokine profiles in 4 day spheroid cultures. Supernatants were collected from spheroids formed by THP-1-derived MΦs or from THP-1-MΦ-JIMT-1 co-cultures, and cytokine secretion was determined using proteome profiling arrays (Figure 7A).

The concentrations of LCN2, MIF, and PAI-1 were found to be particularly elevated in the co-culture spheroids when compared to THP-1-MΦ-only spheroids. A slight increase in CCL2 was detected as well (Figure 7B).

To assess whether these cytokines were regulated by ADP-ribosylation, we treated the spheroids with the PARPi PJ34 and the PARP14i MCD113 and performed RT-qPCR. The results confirmed the data obtained with the cytokine arrays; in fact, the expression of *LCN2*, *MIF*, and *PAI-1* was significantly higher in the spheroid co-cultures than in the THP-1-MΦ-only spheroids (Figure 7C). Moreover, treatments with the ADP-ribosylation inhibitor compounds led to a decreased mRNA expression of these cytokines (Figure 7C).

Our results indicate the involvement of *LCN2*, *MIF*, and *PAI-1* in TAM polarization and suggest a role of PARP14 in the regulation of their gene expression [39,40].

## 3. Discussion

The tumor microenvironment has a great impact on the progression and therapy responsiveness of tumors [12]. Macrophages are the most abundant cell types in various types of cancer, but their presence in the tumor microenvironment is mostly associated with a bad prognosis. Human studies in breast cancer have also shown a correlation between TAMs and poor tumor prognosis [38]. Relatively little is known about how cancer cells reprogram TAMs into a tumor promoting the M2-like phenotype and about the mechanisms of suppression of anti-tumor inflammatory signaling pathways in TAMs. Members of the PARP enzyme family emerged as novel players in the regulation of macrophage polarization [41]. However, their role in shaping the TAM phenotype is not well understood. We therefore aimed to investigate the role of ADP-ribosylation in the polarization of MΦs into the TAM phenotype.

First, we tested the effects of ADP-ribosylation inhibitors PJ34 and MCD113 on IL-4-induced M2 polarization. In line with literature data [42,43], our findings supported the involvement of PARP enzymes in this process. Whereas MCD113 was developed as a PARP14i [30], PJ34 targets primarily PARP1 and -2 but also has a weaker inhibitory effect on PARP14 [28]. Thus, our pharmacological experiments suggested that PARP14 might be involved in M2 polarization. Therefore, we went on to show that PARP14^−/−^ macrophages showed lower IL-4-induced expression of all M2 markers tested, indicating that IL-4-induced M2 polarization is mediated by PARP14. Similar data could be obtained in human MΦs. In our human model, we used six M2 markers, all showing a similar pattern of changes, i.e., induction by IL-4 and inhibition by both PARylation inhibitors.

The above data were also in line with previous reports [20,44], but the role of PARylation in TAMs is less well understood. Therefore, we next set up a cancer-cell-induced TAM polarization with 4T1 mouse breast carcinoma cells and murine BMDMs. The transfer of conditioned media of the tumor cells efficiently reprogrammed MΦs towards an M2-like TAM phenotype. Since both PARPi compounds and the PARP14^−/−^ phenotype significantly reduced the expression levels of M2 markers, we concluded that PARP14 mediates the development of the TAM phenotype. Similar findings could be observed in 3D human spheroids created by co-culturing JIMT-1 breast carcinoma cells and THP-1-derived macrophages. In these co-culture spheroids, the expression of M2 markers was upregulated, while M1 marker expression was reduced, suggesting an M1 to M2 reprogramming. Both PARPi compounds prevented M2 marker gene induction in JIMT-1-THP-1 co-culture spheroids. Our preliminary data (Figure 6) also suggested that PARP inhibition led to the functional reprogramming of macrophages, as indicated by increased apoptosis JIMT-1 cells co-cultured with THP-1-derived macrophages.

We were interested in the potential mediators of MΦ polarization to a TAM phenotype. Several tumor-derived metabolites (e.g., lactate), cytokines, and chemokines [45,46,47,48] have previously been suggested to induce TAM polarization. Here, we used a membrane-based human proteome profiler assay to determine changes in the expression levels of more than hundred human cytokines, chemokines, growth factors, and other soluble proteins in MΦ-tumor cell co-cultures compared to pure MΦ cultures. We identified three proteins displaying higher expression in the co-cultures and reduced expression upon pretreatment with the PARP inhibitor compounds (Figure 7). The MΦ proteins showing PARylation-dependent upregulation in JIMT-1-MΦ co-cultures were lipocalin-2 (LCN2), macrophage migration inhibitory factor (MIF), and plasminogen activator inhibitor-1 (PAI-1).

LCN2 is a multifunctional protein primarily known as a siderophore capable of binding and transporting iron, but it also carries various hydrophobic metabolites (e.g., prostaglandins, steroids, and some hormones) as well. Since dysregulated iron homeostasis and a high demand for iron is an unofficial hallmark of cancer [49,50,51], it is plausible to hypothesize that JIMT-1-induced LCN2 upregulation in macrophages may contribute to TAM polarization. Indeed, several lines of evidence in the literature indicate that high levels of LCN2 are associated with a bad prognosis and therapy resistance of breast cancer [52,53,54]. Moreover, LCN2 is also known to associate with MMP9, preventing the degradation of the metalloproteinase that is involved in degrading ECM, enhancing cancer metastasis [55]. However, this latter scenario is unlikely to be important in our model as LCN2 upregulation was accompanied by MMP9 downregulation (Figure 7B). The way in which the PARP inhibitors suppressed *LCN2* expression requires further investigation. It is possible that both compounds exerted their effects through PARP14 inhibition, but considering the role of the *NFκB* pathway in the regulation of *LCN2* expression and the *NFκB* co-activator role of PARP1, the effect of PJ34 could also be through the inhibition of *NFκB* signaling.

MIF emerged as another potential mediator of the PARylation-dependent TAM polarization. Like LCN2, MIF was also induced in MΦs by JIMT-1 cells, and this induction was reduced by both PARPi compounds. *MIF1* has previously been implicated in M2 polarization of MΦs, as demonstrated in the MΦ of glioblastoma, cisplatin-resistant lung cancer, and oral squamous cell carcinomas [56,57,58]. Furthermore, cytoplasmic MIF has also been proposed to have prognostic value in squamous cell carcinoma patients [59]. Considering these findings, it is quite likely that the inhibition of *MIF* expression by the PARPis may contribute to the reversion of the TAM phenotype in breast cancer.

The serine protease inhibitor PAI-1 plays a role in the recruitment of MΦs to the tumors [60]. In addition, it is also an important macrophage-derived mediator of cancer progression [61]. It has been shown to be involved in M2 polarization in various tumor-related settings [61,62,63]. Therefore, PARylation-dependent PAI-1 induction is likely to play a role in TAM polarization and may thus represent a potential target in breast cancer treatment.

Based on the observations detailed above, we believe that is worth following up on the role of PARylation in the regulation of *LCN2*, *MIF*, and *PAI-1* expression as it may contribute to the anticancer effect of PARylation of various different types of cancer with high abundance of infiltrating MΦs.

An important question is the role of different PARP family members in the development of the TAM phenotype. Our current data underline the importance of PARP14, not only in IL-4-induced M2 but also in cancer-cell-induced TAM polarization. Others also reported that targeting PARP14 shifts macrophages from an M2 to an M1 phenotype [20,64]. The role of PARP1 in TAM polarization cannot be excluded either. This scenario is supported by findings that the PARP1/2-specific, clinically used PARPi Olaparib inhibits tumor growth, and this effect is abolished when MΦs are depleted [65]. However, Wang’s group demonstrated that Olaparib-induced macrophage reprogramming takes place also in the absence of PARP1 or PARP2 [27]. Thus, it seems plausible to hypothesize that depending on the specifics of the model, both PARP14 and PARP1/2 contribute to TAM polarization.

An interesting corollary to our findings is that preventing the development of the TAM phenotype (e.g., by PARP14 inhibition) may also increase the efficacy of the PARPi treatment of BRCA mutant breast cancer. TAMs have been reported to contribute to PARPi resistance [44], and reprogramming TAMs (e.g., by STING agonism) is effective in restoring the PARPi sensitivity of breast cancer [66]. This may imply that PARPis targeting both DNA-damage-responsive PARPs (PARP1 and 2) and PARP14 may possess increased antitumor potential in breast cancer.

In conclusion, our data support a scenario in which breast-cancer-derived signals induce TAM polarization in macrophages. Our findings show the effectiveness of PARPis in the downregulation of protumor genes, highlighting the importance of a better understanding in the switching mechanisms of TAMs from M2 into M1 phenotype as a potential effective treatment of cancers [67]. PARylation is essential for the development of the TAM phenotype, with PARP14 reported to play a dominant role in this process [20]. Our findings suggest that targeting PARP14 may have a therapeutic potential in breast cancer by reprogramming tumor-growth-promoting macrophages towards an anti-cancer phenotype. It remains to be seen if PARP14 also plays similar roles in other types of cancer and whether the reversal of the TAM phenotype by PARP14 inhibition effectively unleashes anti-tumor effector mechanisms of the adaptive immune system.

## 4. Materials and Methods

### 4.1. Materials

Dulbecco’s modified Eagle’s medium-high glucose (DMEM, D6429) and recombinant mouse IL-4 (l1020) were purchased from Sigma-Aldrich (Budapest, Hungary). Fetal bovine serum (FBS, FB-1090/500) and penicillin–streptomycin (LM-A4118/100) were from Biosera (Cholet, France). PARP14^−/−^ mice [31] were from the Boothby Lab (Vanderbilt University, Nashville, TN, USA). Recombinant mouse M-CSF (416-ML-010/CF) was purchased from R&D Systems (Minneapolis, MN, USA). PJ34 (2-(dimethylamino)-N-(6-oxo-5H-phenanthridin-2-yl)acetamide hydrochloride, 344458-15-7) was obtained from Sigma-Aldrich (Budapest, Hungary), and MCD113 was synthesized by Dana Ferraris’s group at McDaniel College (Westminster, MD, USA), as previously reported [30]. (In the cited reference, MCD113 was referred to as compound 4t, and the IC_50_ of the inhibitor was 160 nM for PARP14.) L-glutamine (BEBP17-605E) and PBS (BE17-517Q) were from Lonza (Basel, Switzerland). Ficoll-Paque Plus (17-1440-03) was obtained from Amersham Biosciences (Amersham, UK). Anti-CD14-conjugated microbeads (130-050201) were from Miltenyi Biotec (Bergisch Gladbach, Germany), and RPMI (R5886-500ML) was from Sigma-Aldrich (Budapest, Hungary). FCS (10270-106) and antibiotic/antimycotic solution (15140-122) were from Gibco (Waltham, MA, USA). Human M-CSF (300-25) and IL-4 (200-04) were purchased from PeproTech (Waltham, MA, USA). Phorbol 12-myristate 13-acetate (PMA, P1585), DMEM/F-12 (D8437), and human IFN-γ (13255) were purchased from Sigma-Aldrich (Budapest, Hungary). RPMI 1640 (L0501-500) was from Biowest (Nuaillé, France), and insulin Humulin R (HI0219) was obtained from Eli Lilly Canada Inc. (Toronto, ON, Canada). TRI Reagent (TR118) was from the Molecular Research Center (Cincinnati, OH, USA), the High Capacity cDNA Reverse Transcription Kit (4668814) was from Applied Biosystems (Foster City, CA, USA), and 2× qPCRBIO Sygreen Mix Lo-ROX (PB20.11-50) was from PCR Biosystems (London, UK). Oligonucleotides were purchased from IDT (Coralville, IA, USA) or Sigma-Aldrich (St. Louis, MO, USA). Anti-CD206-allophycocyanin (APC 550889) antibody conjugate was from BioLegend (San Diego, CA, USA). 7-Aminoactinomycin-D (7-AAD, A1310) and pluronic-F127 (P2443) were obtained from Sigma-Aldrich (Budapest, Hungary). Cell Tracker Blue (CTB, 1856665) and Annexin V-Alexa Fluor 647 conjugate (A23204) were purchased from Invitrogen-ThermoFisher Scientific (Waltham, MA, USA). Agarose (A9539) and dimethyl sulfoxide (DMSO, D8418) were from Sigma (Budapest, Hungary). Glass-bottom Cell Carrier-96 ultra microplates (6055300) were from PerkinElmer (Waltham, MA, USA). The Proteome Profiler Human XL Cytokine Array Kit (ARY022B) was from R & D Systems (Minneapolis, MN, USA), and the IRDye 800CW Streptavidin (926-32230) was from LI-COR (Lincoln, NE, USA).

### 4.2. Cell Lines

All cell lines were cultured under standard conditions (humidified atmosphere, 5% CO_2_ at 37 °C) and were routinely checked for the absence of mycoplasma contamination.

4T1 mouse mammary tumor cells were cultured in DMEM-high glucose (Sigma-Aldrich, D6429), supplemented with 10% FBS (Biosera, FB-1090/500), 5% L-glutamine (Lonza, BEBP17-605E), and 5% penicillin–streptomycin (Biosera, LM-A4118/100) at 37 °C in a humidified atmosphere containing 5% CO_2_.

THP-1 human monocytic cells were cultured in RPMI 1640 (Biowest, L0501-500) supplemented with 10% FBS (Biosera, FB-1090/500) and 5% penicillin/streptomycin (Biosera, LM-A4118/100).

JIMT-1 and JIMT-1-Enhanced Green Fluorescence Protein (EGFP) breast cancer cells were cultured in DMEM/F-12 (Sigma, D8437), supplemented with 20% FBS (Biosera, FB-1090/500), 0.3 U/mL insulin (100 NE/mL, Humulin R, HI0219), and 1% penicillin–streptomycin (Biosera, LM-A4118/100). An EGFP expressing subline (JIMT-1-EGFP) was generated as previously described (Guti E. et al., 2022 [36]) and was used for high-content analysis (HCA).

### 4.3. Murine Bone-Marrow-Derived Macrophages (BMDMs)

Bone marrow cells were isolated from 10–12-week-old male C57/BL6 mice or from PARP14^−/−^ mice [30] and their PARP14^+/+^ counterparts as previously described [68]. Mice were kept in the laboratory animal facility of the University of Debrecen under normal room temperature and had access to water and standard lab chow ad libitum. Collection of mouse bone marrow MΦs was approved by the Regional Research Ethics Committee (15/2016/DEMÁB). Cells were seeded at a density of 10^6^/mL into tissue culture plates in Dulbecco’s modified Eagle’s medium-high glucose (DMEM, Sigma-Aldrich, D6429), supplemented with 10% heat-inactivated fetal bovine serum (FBS, Biosera, FB-1090/500), 5% L-glutamine (Lonza, BEBP17-605E), and 5% penicillin–streptomycin (Biosera, LM-A4118/100). Cells were maintained at 37 °C and 5% CO_2_. Differentiation was induced with 25 ng/mL recombinant mouse M-CSF (R&D Systems, 416-ML-010/CF) on day 3 and day 5 of culture. On day 6, cells were used as fully differentiated bone-marrow-derived MΦs (BMDMs). BMDMs were polarized for 1 day with 5 ng/mL recombinant mouse IL-4 (Sigma-Aldrich, l1020) to M2 MΦs or with 4T1 conditioned media (4T1CM) to TAMs (see details below). Cells were washed with PBS (Lonza, BE17-517Q) and subsequently collected for further experiments.

### 4.4. Preparation of 4T1 Conditioned Media (4T1CM) and Induction of the TAM Phenotype

4T1 cells were maintained in full DMEM-high glucose culture medium as specified in Section 4.2 above. Once reaching approximately 80% confluency, 4T1 cells were seeded into 6-well tissue culture plates at a density of 10^6^/mL in culture media and incubated for 1 day at 37 °C. Thereafter, cell culture supernatant was cautiously collected from 4T1 cells, centrifuged at 150 RCF for 3 min at room temperature to remove cell debris, and added to BMDM cells for polarization into TAMs (note: the IL-4 content of 4T1CM was quantitated with bead assay (BD CBA Mouse IL-4 Flex Set; BD Biosciences, Cat. No.: 558298) and was found to contain no IL-4 protein). 4T1-conditioned media was diluted at a 1:1 ratio [32,33] with full DMEM and added to BMDMs. BMDMs were incubated for 24 h and were then washed with PBS (Lonza, BE17-517Q) and collected for further experiments.

### 4.5. Human Peripheral Blood Mononuclear Cells (PBMCs)

Heparinized leukocyte-enriched buffy coats were obtained from healthy blood donors drawn at the Regional Blood Center of the Hungarian National Blood Transfusion Service (Debrecen, Hungary) in accordance with the written approval of the Director of the National Blood Transfusion Service and the Regional and Institutional Research Ethical Committee of the University of Debrecen, Faculty of Medicine, Debrecen, Hungary (protocol number OVSzK 3572-2/2015/5200). Written informed consent was obtained from the blood donors prior to blood donation. Peripheral blood mononuclear cells (PBMCs) were separated from buffy coats by Ficoll-Paque Plus (Amersham Biosciences, 17-1440-03) gradient centrifugation. According to the manufacturer’s protocol, monocytes were purified from PBMCs by positive selection using immunomagnetic anti-CD14-conjugated microbeads. After separation on a VarioMACS magnet, 96–99% of the cells were shown to be CD14^+^ monocytes, as measured by flow cytometry. Isolated monocytes were cultured for 5 days in 24-well tissue culture plates at a density of 1.5 × 10^6^ cells/mL in RPMI supplemented with 10% FCS (Gibco, 10270-106) and 1% antibiotic/antimycotic solution (Gibco, 15140-122) in the presence of 50 ng/mL human M-CSF (PeproTech, 300-25) for MΦ polarization. The medium was refreshed and complemented with 50 ng/mL M-CSF on day 2 of differentiation. On day 5, cells were further cultured for 24 h in the presence of 100 ng/mL human IL-4 (PeproTech, 200-04) in RPMI supplemented with 10% FCS and 1% antibiotic/antimycotic solution to induce M2 polarization.

### 4.6. THP-1 MΦ Differentiation

To induce differentiation into MΦs, THP-1 cells were seeded into a 6 cm Petri dish at 1.5 × 10^6^/mL seeding density and incubated with 1 ng/mL PMA for 48 h (M0 MΦs) in full RPMI 1640 media. Cells were then gently detached from the Petri dish using a cell scraper, centrifuged at 150 RCF for 3 min at room temperature, and used for the generation of spheroids (see details below).

### 4.7. Generation of THP-1-Derived MΦ Spheroids, JIMT-1 Spheroids, and JIMT-1-THP-1 Co-Culture Spheroids

Spheroids were grown as previously described [69] with modifications as follows. We pre-coated 96-well cell culture plates with 0.5% agarose (Sigma, A9539)-PBS solution (30 µL/well) to form a U-shaped, cell-repellent bottom. For THP-1-derived MΦ and JIMT-1 spheroids, cells were seeded at a 2 × 10^4^/mL density in 100 μL (2000 cells/spheroid) into pre-coated wells of 96-well plates in fully supplemented DMEM/F-12 JIMT-1 media. Cells were allowed to clump together for 3 days. For co-culture spheroids, THP-1-cell-derived M0 MΦs were mixed at a 1:1 ratio with JIMT-1 cells (see details below). The ratio was optimized to better represent the infiltration of immune cells in the tumor and the in vivo situation [37]. Each of the two cell populations was seeded at a density of 2 × 10^4^/mL into the wells of pre-coated 96-well plates in a volume of 50 μL, and cells were allowed to clump together as described above. Specifically, a total number of 4000 cells were seeded for each spheroid (2000 cells/cell type) in a total volume of 100 µL/well in JIMT-1 growth media. On day 3 after the induction of spheroids, for further MΦ polarization to M1, 20 ng/mL recombinant human IFN-γ was added to the spheroids for another 24 h. Spheroids were transferred to 15 mL Falcon tubes and centrifuged at 150 RCF for 3 min at room temperature. The cell pellet was collected for further experiments.

### 4.8. RNA Isolation, Reverse Transcription, and Quantitative Real-Time PCR

Total RNA was isolated from BMDM cells, human MΦs, and spheroids using the TRI Reagent. A total of 1 μg RNA was used for reverse transcription, and cDNA synthesis was performed using the High Capacity cDNA Reverse Transcription Kit (Applied Biosystems, 4668814) according to the manufacturer’s instructions. cDNA samples were used for quantitative real-time PCR analysis with 2× qPCRBIO Sygreen Mix Lo-ROX (PCR Biosystems, PB20.11-50), and PCR was performed in a total volume of 10 µL in triplicate on a Roche Lightcycler 480II (Basel, Switzerland). Oligonucleotides were purchased from IDT (Coralville, IA, USA) or Sigma-Aldrich (St. Louis, MO, USA). The mouse and human primer pair sequences are presented in Tables S1 and S2, respectively. Fold changes in expression were calculated by the ΔΔCt method using geometrical means of a combination of housekeeping genes as an endogenous control for mRNA expression (mouse *HPRT*, *B2M*, *GAPDH*, *Cyclophilin A*, and *36B4* and human *GAPDH*, *Cyclophilin A*, and *36B4*). All fold changes are expressed as normalized to the untreated control.

### 4.9. Flow Cytometry

Phenotyping of human monocyte-derived MΦs was performed by flow cytometry using anti-CD206-allophycocyanin antibody conjugate. Cell viability was assessed by 7-aminoactinomycin-D (7-AAD, Sigma-Aldrich, A1310) staining. Samples were stained for 10 min with 10 μg/mL 7-AAD immediately before flow cytometric analysis. Fluorescence intensities and the frequency of CD206-expressing cells were measured with a Novocyte2000R Flow Cytometer (Agilent (Acea) Biosciences Inc., San Diego, CA, USA), and data were analyzed with the FlowJo v X.0.7 software (Tree Star, Ashland, OR, USA).

### 4.10. High-Content Analysis (HCA) on Live Cells: Cell Death in Spheroids

For all HCA experiments, co-culture spheroids were generated using THP-1-derived MΦs and JIMT-1-enhanced green fluorescent protein (EGFP) cells. In order to distinguish between the two cell populations, M0 THP-1-derived MΦs (differentiated as described above) were stained before seeding with 10 µM Cell Tracker Blue (CTB; Invitrogen, 1856665) for 1 h in THP-1-growth media (see composition above). Cells were then washed twice with RPMI serum-free media (Biowest, L0501-500) to eliminate the excess of the dye (150 RCF for 3 min at room temperature). Stained M0 cells were seeded into 96-well plates previously coated with 0.5% agarose in PBS and mixed at a 1:1 ratio with JIMT-1-EGFP cells in JIMT-1 media (2 × 10^4^/mL for each cell population in a total volume of 100 μL, as detailed above). On day 3, spheroids were then transferred in triplicates to glass-bottom Cell Carrier-96 ultra microplates (PerkinElmer, 6055300). In order to prevent cell attachment to the bottom of the wells, plates were pre-coated with 0.5% pluronic-F127 (Sigma, P2443) in DMSO (Sigma, D8418) for 45 min at room temperature (50 µL/well), and wells were washed twice with 100 µL DMEM/F-12-serum free media. Human IFN-γ (20 ng/mL, Sigma, 13255) was added to the spheroids for 24 h to induce MΦ polarization to M1. Cells were stained with Annexin V-Alexa Fluor 647 conjugate (Invitrogen-ThermoFisher Scientific, A23204) for 1 h in JIMT-1 growth media to detect apoptotic cell death (1:100 in 50 µL/well). After staining, images were taken with Opera Phenix High-Content Analysis equipment (Perkin Elmer, Waltham, MA, USA), and data were analyzed with the Harmony 4.9 software (Perkin Elmer). Specifically, images of three spheroids/condition were taken (10× air objective with 0.3 numerical aperture in confocal mode) and analyzed for spheroid area and for the intensity of Annexin V 647 in tumor cells in each well. Fluorescence intensity was detected at the following channels: EGFP (ex: 488 nm, em: 500–550 nm), Alexa647 (ex: 640 nm, em: 650–760 nm), and DAPI (ex: 405nm, em: 435–480 nm). Spheroids were identified by the EGFP fluorescence of JIMT-1 cells by using the “Find Texture Regions” option, and they were filtered out by size (>25,000). Red cells (Annexin-positive cells) in green (JIMT-1-EGFP cells) were selected by the “Select Population” option and were identified as apoptotic tumor cells, being measured for the Annexin V fluorescence intensity in each whole well. Annexin intensities were determined and expressed as mean intensity.

### 4.11. Human Cytokine Array

The THP-1-derived MΦ spheroids and JIMT-1-MΦ spheroid co-cultures were collected, and soluble proteins were detected using the Proteome Profiler Human XL Cytokine Array Kit (R&D Systems, ARY022B) in accordance with the manufacturer’s guidelines. IRDye 800CW Streptavidin (LI-COR, 926-32230) was added to the membranes, and for imaging, the arrays were scanned using a LI-COR Odyssey Infrared Imaging System (LI-COR Biosciences, Lincoln, NE, USA).

### 4.12. Statistical Analysis

Data are presented as mean ± SEM of at least three independent experiments. The Shapiro–Wilk test was used to analyze normality. The Kruskal–Wallis test followed by Dunn’s post-hoc test was performed when the distribution of data was not normal. If the data distribution was normal, one-way ANOVA complemented by Tukey’s, Dunnett’s, or Bonferroni’s post-hoc test was used. Statistical analysis was performed using GraphPad Prism 9.5.1 (GraphPad Software Inc., San Diego, CA, USA). *p* values < 0.05 were considered significant, as stated in figure legends.

## Data Availability

The data presented in this study are available on reasonable request from the corresponding author. The data are not publicly available due to privacy or ethical restrictions.

## Supplementary Materials

The following supporting information can be downloaded at https://www.mdpi.com/article/10.3390/ijms25073601/s1.
