# Supplementary material for: PARP14 Contributes to the Development of the Tumor-Associated Macrophage Phenotype
Source: Int J Mol Sci. 2024 Mar 22;25(7):3601. doi: 10.3390/ijms25073601 (PMC11011797; doi:10.3390/ijms25073601)
Supplement: Supplementary file 1 [file ijms-25-03601-s001.zip › Supplementary Tables.pdf]

Supplementary Tables

Supplementary Table S1. List of mouse primers used for gene expression analysis by quantitative real-time PCR.

Gene name	Forward primer	Reverse primer
**mArg1**	CTTGCGAGACGTAGACCCTG	GGCCTTTTCTTCCTTCCCAG
**mFizz-1**	GCCAATCCAGCTAACTATCCCT	CAACGAGTAAGCACAGGCAG
**mMRC1**	GCAAATGGAGCCGTCTGTGC	CTCGTGGATCTCCGTGACAC
**mHPRT**	AGTGTTGGATACAGGCCAGAC	CGTGATTCAAATCCCTGAAGT
**mB2M**	CTGCTACGTAACACAGTTCCACCC	CATGATGCTTGATCACATGTCTCG
**mGAPDH**	ACAGTCCATGCCATCACTGCC	GCCTGCTTCACCACCTTCTTG
**mCyclophilin A**	TCCGACTGTGGACAGCTCTA	ATTGCGAGCAGATGGGGTAG
**m36B4**	AAGCGCGTCCTGGCATTGTCT	CCGCAGGGGCAGCAGTGGT

Supplementary Table S2. List of human primers used for gene expression analysis by quantitative real-time PCR.

Gene name	Forward primer	Reverse primer
**hCD-14**	GGAAGACTTATCGACCATGGA	GCTGAGGTTCGGAGAAGTTG
**hIL-12**	CCAAGAACTTGCAGCTGAAG	TGGGTCTATTCCGTTGTGTC
**hTNFa**	TGGGTCTATTCCGTTGTGTC	GAGGGCTGATTAGAGAGAGGTC
**hARG1**	TCATCTGGGTGGATGCTCACAC	GAGAATCCTGGCACATCGGGAA
**hRETNLB (Fizz-1)**	GCAAGAAGCTCTCGTGTGCTAG	AACATCCCACGAACCACAGCCA
**hMRC1**	CGAGGAAGAGGTTCGTTCACC	GCAATCCCGGTTCTCATGGC
**hFibronectin**	CCACCCCCATAAGGCATAGG	GTAGGGGTCAAAGCACGAGTCAT
**hCCL17**	ACTTCAAGGGAGCCATTCCC	CCCTGCACAGTTACAAAA
**hCCL18**	CTCTGCTGCCTCGTCTATACCT	CTTGGTTAGGAGGATGACACCT
**hLCN2 (Lipocalin2)**	GTGAGCACCAACTACAACCAGC	GTTCCGAAGTCAGCTCCTTGGT
**hMIF**	AGAACCGCTCCTACAGCAAGCT	GGAGTTGTTCCAGCCCACATTG
**hPAI-1 (Serpin E1)**	CTCATCAGCCACTGGAAAGGCA	GACTCGTGAAGTCAGCCTGAAAC
**hGAPDH**	AGCCTCAAGATCAGCAATG	ATGGACTGTGGTCATGAGTCCTT
**hCyclophilin A**	GTCTCCTTTGAGCTGTTTGCAGAC	CTTGCCACCAGTGCCATTATG
**h36B4**	CCATTGAAATCCTGAGTGATGTG	GTCGAACACCTGCTGGATGAC
