# Supplementary figures and images for: PARP14 Contributes to the Development of the Tumor-Associated Macrophage Phenotype
Source: Int J Mol Sci. 2024 Mar 22;25(7):3601. doi: 10.3390/ijms25073601 (PMC11011797; doi:10.3390/ijms25073601)

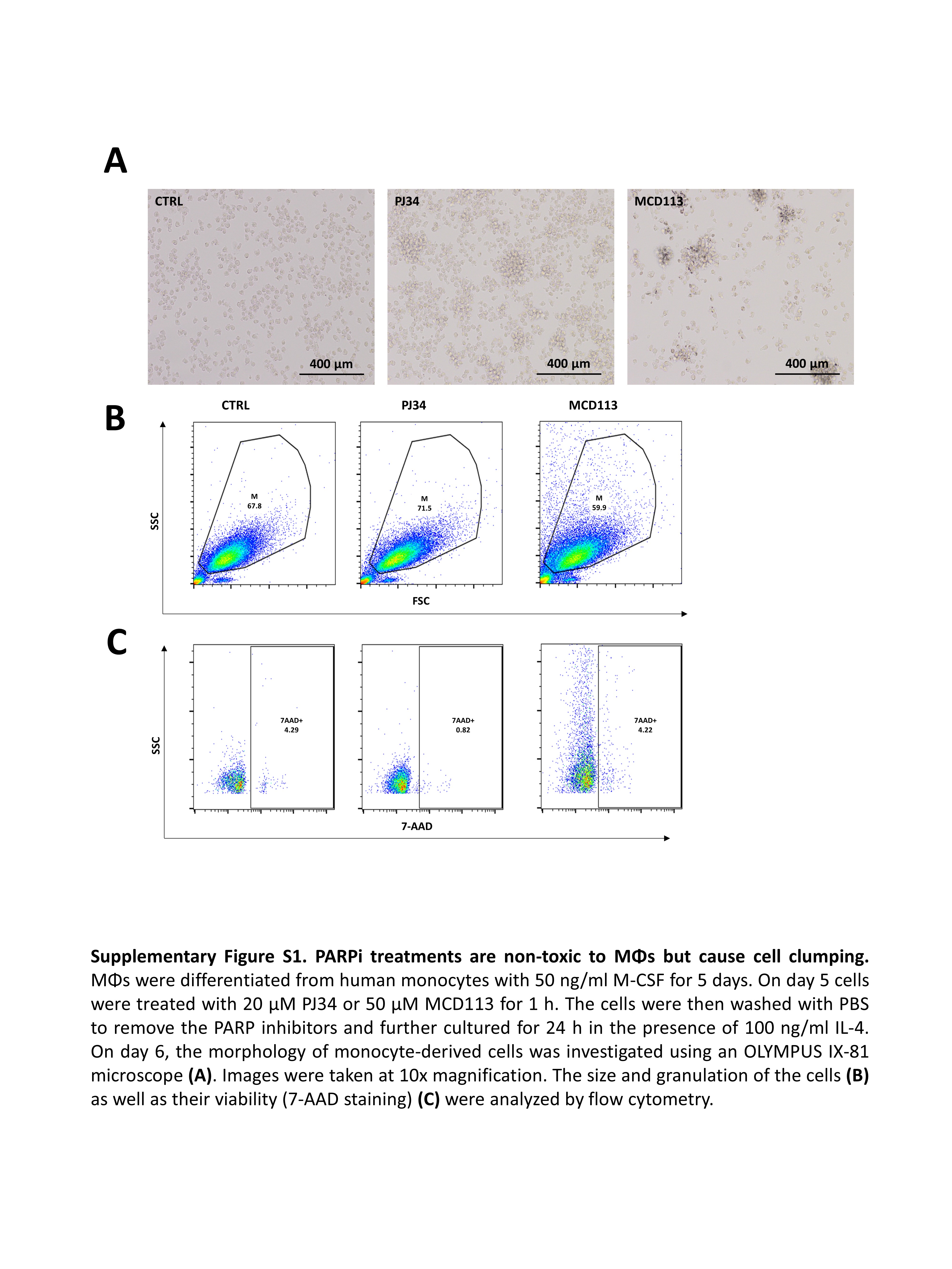

Supplement: Supplementary file 1 [file ijms-25-03601-s001.zip › Supplementary Figure S1.jpg]

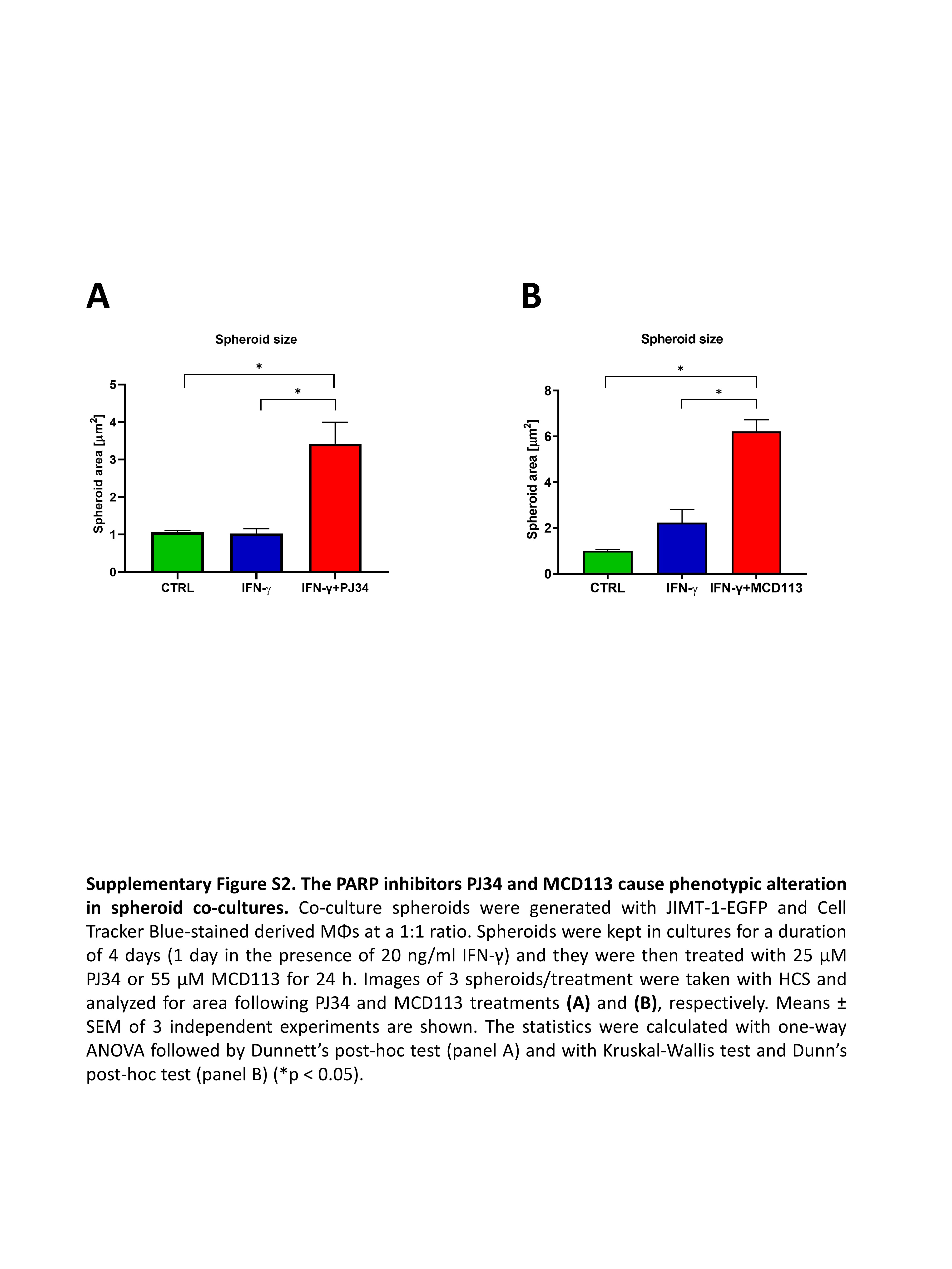

Supplement: Supplementary file 1 [file ijms-25-03601-s001.zip › Supplementary Figure S2.jpg]
